# Cardiovascular-renal protective effect and molecular mechanism of finerenone in type 2 diabetic mellitus

**DOI:** 10.3389/fendo.2023.1125693

**Published:** 2023-02-13

**Authors:** Ruolin Lv, Lili Xu, Lin Che, Song Liu, Yangang Wang, Bingzi Dong

**Affiliations:** ^1^ Department of Endocrinology and Metabolism, The Affiliated Hospital of Qingdao University, Qingdao, China; ^2^ Department of Nephrology, The Affiliated Hospital of Qingdao University, Qingdao, China; ^3^ Department of Cardiology, The Affiliated Hospital of Qingdao University, Qingdao, China

**Keywords:** T2DM, finerenone, mineralocorticoid receptor antagonists, chronic kidney disease, cardiorenal protection

## Abstract

Chronic kidney diseases (CKD) and cardiovascular diseases (CVD) are the main complications in type 2 diabetic mellitus (T2DM), increasing the risk of cardiovascular and all-cause mortality. Current therapeutic strategies that delay the progression of CKD and the development of CVD include angiotensin-converting enzyme inhibitors (ACEI), angiotensin II receptor blockers (ARB), sodium-glucose co-transporter 2 inhibitors (SGLT-2i) and GLP-1 receptor agonists (GLP-1RA). In the progression of CKD and CVD, mineralocorticoid receptor (MR) overactivation leads to inflammation and fibrosis in the heart, kidney and vascular system, making mineralocorticoid receptor antagonists (MRAs) as a promising therapeutic option in T2DM with CKD and CVD. Finerenone is the third generation highly selective non-steroidal MRAs. It significantly reduces the risk of cardiovascular and renal complications. Finerenone also improves the cardiovascular-renal outcomes in T2DM patients with CKD and/or chronic heart failure (CHF). It is safer and more effective than the first- and second-generation MRAs due to its higher selectivity and specificity, resulting in a lower incidence of adverse effects including hyperkalemia, renal insufficiency and androgen-like effects. Finerenone shows potent effect on improving the outcomes of CHF, refractory hypertension, and diabetic nephropathy. Recently studies have shown that finerenone may have potential therapeutic effect on diabetic retinopathy, primary aldosteronism, atrial fibrillation, pulmonary hypertension and so on. In this review, we discuss the characteristics of finerenone, the new third-generation MRA, and compared with the first- and second-generation steroidal MRAs and other nonsteroidal MRAs. We also focus on its safety and efficacy of clinical application on CKD with T2DM patients. We hope to provide new insights for the clinical application and therapeutic prospect.

## 1 Introduction

Type 2 diabetes mellitus (T2DM) is a metabolic disorder characterized by hyperglycemia with insulin resistance. The management of T2DM requires multifactorial behavioral and pharmacological treatments to prevent or delay complications, and improves the quality of life. CKD and CVD are the common complications of T2DM (1). Among patients with T2DM, cardiovascular complications are the leading cause of morbidity and mortality, and kidney complications are highly prevalent in patients with T2DM (2). Available therapeutic strategies that delay the progression of CKD and the development of CVD include angiotensin-converting enzyme inhibitors (ACEI), angiotensin II receptor blockers (ARB), sodium-glucose co-transporter 2 inhibitors (SGLT-2i) and GLP-1 receptor agonists (GLP-1RA).

Finerenone is a structurally novel non-steroidal mineralocorticoid receptor antagonist (MRAs), which exhibits outstanding effect on cardio-renal protection (3). The mineralocorticoid receptors (MRs) are widely distributed in the heart, kidney, brain, lung, colon, skin, liver, skeletal muscle, saliva, sweat gland, and fat (4). MRs are mainly expressed in the cardiovascular system and kidney, and play vital role in ventricular remodeling and chronic heart failure (CHF) progression (5). Aldosterone, the MR, maintains the sodium/potassium homeostasis and the electrolyte balance of the body. In addition, an increasing number of studies have shown that inflammatory and fibrotic effect is mediated by excessive activation of MRs, leading to the adverse cardiac and renal outcomes. It could be an important therapeutic target for chronic kidney disease (CKD) induced by T2DM. Finerenone, a third-generation highly selective MRA, can directly and specifically block MR hyperactivation, and promote the anti-inflammatory and anti-fibrotic effects. In this way, finerenone exhibits cardiovascular and renal double-benefits, and is used in the treatment of T2DM-related CKD (diabetic kidney disease, DKD) to reduce the risk of persistent decline in glomerular filtration rate (eGFR) and the progression of end stage renal disease (ESRD). In general, finerenone could reduce the risk of cardiovascular and renal outcomes (3, 6).

A number of large-scaled clinical trials have proved that finerenone can significantly reduce both cardiorenal endpoints and the adverse reactions such as electrolyte disorders and sex hormone-like effects (7). In this review, we discuss the pharmacological characteristic, molecular mechanism, effectiveness and safety of finerenone in the treatment of T2DM with CKD/DKD and CVD, to provide clinical evidence and deep insight for therapeutic strategies.

## 2 The mechanism of MR activation on kidney and cardiovascular system

### 2.1 The physiological action of MR and MRAs

The physiological ligands of MR are mainly aldosterone and cortisol. MR is expressed in a variety of tissues and cells, including cardiomyocytes, vascular endothelial cells, vascular smooth muscle cells, renal tubular epithelial cells and macrophages (4). Aldosterone binds to MR in the distal renal tubular epithelial cells to form aldosterone-MR complex, which promotes the reabsorption of sodium and excretion of potassium and hydrogen ions, suggesting MR plays an important physiological role in the regulation of water and salt balance, blood pressure and circulating blood volume (8).

### 2.2 The pathological effect and mechanism of MR activation

MR is also involved in the inflammatory response, regulating the expression of cytokines and inflammatory mediators, the activation of the inflammatory pathways and infiltration of inflammatory cells (9). Excessive activation of MR promotes reactive oxygen species (ROS) production, mediates the inflammatory and fibrogenic processes, and ultimately leads to myocardial hypertrophy and ventricular remodeling (10), as well as the renal damage, glomerular hypertrophy, glomerulosclerosis, and vascular damage such as vascular endothelial dysfunction and vascular smooth muscle cells proliferation (5). MR overactivation act directly on vascular smooth muscle cells *via* the MR-VEGFR1 pathway, leading to cell proliferation and enhanced vascular fibrosis, thickness and stiffness. In addition, MR overactivation also promotes the differentiation of inflammatory cells such as macrophages, T lymph cells into a pro-inflammatory phenotype in mice model. It further promotes the chronic inflammation microenvironment, and damages target organs and accelerates the disease process (11). MR overactivation results in renal injury and mineralocorticoid sensitive hypertension directly through the MR-Rac1 pathway, and cause glomerular hyperfiltration. In animal study, MR gene knockdown in cardiovascular endothelial cells improves renal inflammation and fibrosis by reducing inflammatory macrophage differentiation and inhibiting the expression of inflammatory and fibrosis-related genes (12).

Therefore, blocking MR over-activation could be beneficial to improve target organ damage.

## 3 Cardio-renal protective mechanism of finerenone

### 3.1 Moderating mechanism of finerenone on cardiovascular protection

**Figure 1:**
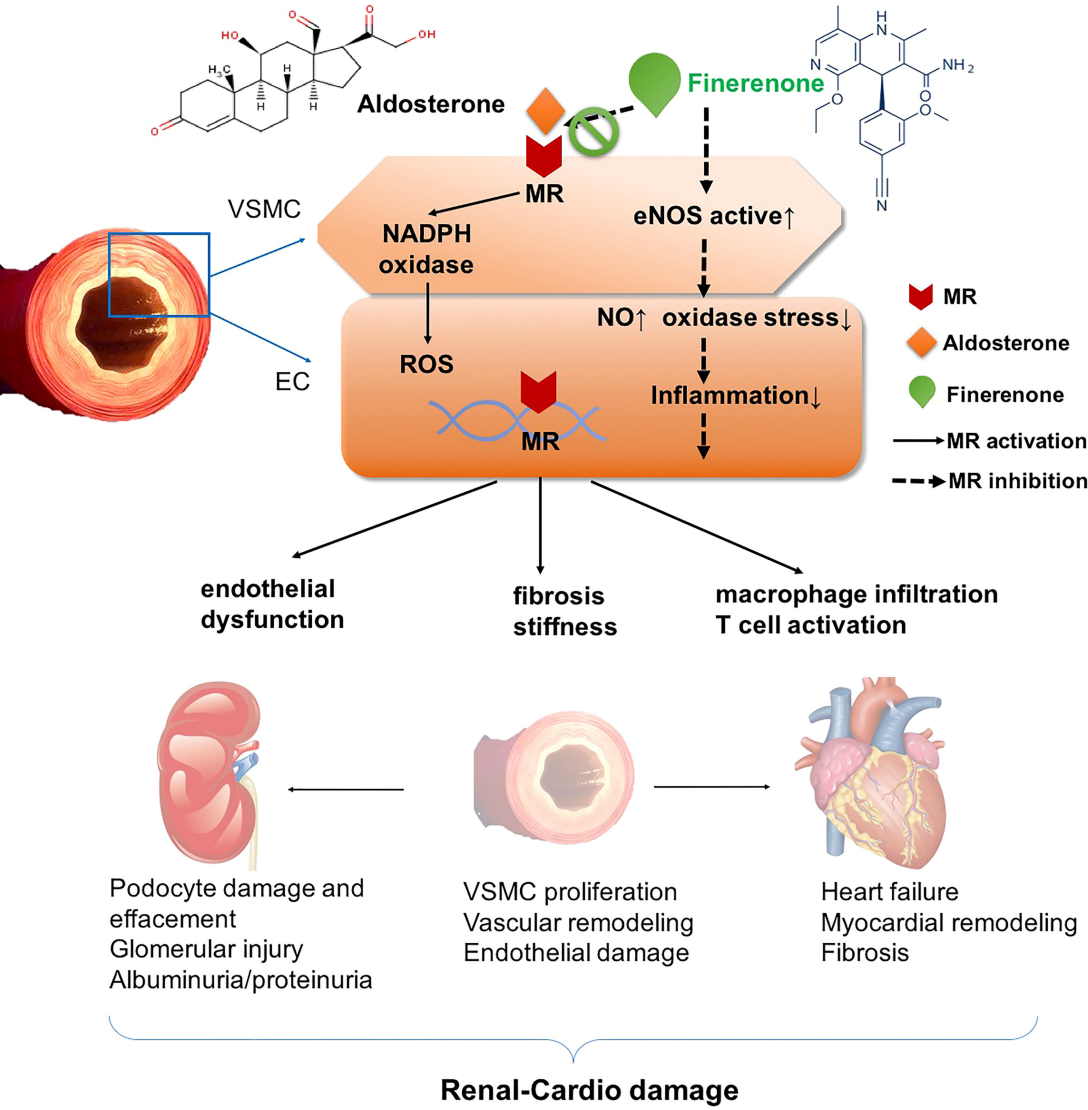
Mechanism of renal-cardio damage by mineralocorticoid receptor (MR) overactivation. MR activation plays important role in promoting NADPH oxidase, and enhancing ROS accumulation in VSMCs and ECs, and induces oxidase stress. In this way, MR agonist such as aldosterone results in endothelial dysfunction, macrophage infiltration and T cell activation, inflammatory progenitors including cytokines collection, and acts on VSMC leading to fibrosis and stiffness. MR activation affects kidney, aggravates podocyte damage and effacement, glomerular injury, and VSMC proliferation and endothelial damage, leading to vascular remodeling. On heart, MR activation exacerbates heart failure, myocardial remodeling and fibrosis. On contrast, MR antagonist finerenone blocks the binding of aldosterone and MR, then attenuates those pathophysiological progressions. In this way, finerenone shows renal-cardio protective effect. MR, mineralocorticoid receptor. EC, endothelial cells. VSMC, vascular smooth muscle cells. ROS, reactive oxygen species. NADPH, nicotinamide adenine dinucleotide phosphate.

CVD is a common co-morbidity of T2DM. MR overactivation plays an important role in the cardiovascular progression of T2DM with CVD. The mechanism of MR overactivation involving in cardiovascular damage is as follows. (1) MR overactivation increases NADPH oxidase activity to induce a series of oxidative stress responses in adult rat models, leading to the inflammatory and fibrotic process, finally results in the cardiac lesions such as myocardial hypertrophy, ventricular remodeling, myocardial ischemia/infarction, and ultimately to the development and progression of cardiovascular disease and renal disease (13, 14). (2) In addition, high aldosterone levels cause water and sodium retention and sodium overload, and increase production of ROS, thus exerting inflammatory reaction, fibrotic progression and oxidative stress (15). Those factors act on the heart result in remodeling of the heart and arteries, triggering the risk of decreased left heart function, ventricular remodeling, and arrhythmias, all can deteriorate myocardial infarction and heart failure (HF) (12). (3) Furthermore, MR activation leads to vascular smooth muscle cell proliferation, increases vascular stiffness through vascular endothelial growth factor receptor 1 (VEGFR1), worsens vascular injury by decreasing nitric oxide, disturbs vascular endothelial dysfunction and vasoconstriction in rats (16). Therefore, targeted blockade of MR overactivation can ameliorate the inflammatory and fibrotic injury mediated by this pathway (17) (**Figure 1**). It is a key therapeutic target for patients with T2DM-related CVD.

MRAs promote co-factor SRC-1 recruitment to an MR-dependent promoter. The third-generation MRA finerenone has highly potent and selectivity for MR. Compared to spironolactone, finerenone binds to MR in a manner of unstable receptor-ligand complex, and leads to less recruit co-regulators (18). Finerenone delays the nuclear accumulation of MR-aldosterone complex, and blocks the recruitment of critical transcription cofactors. Thus, finerenone disturbs the steps downstream of MR pathway, and decreased expression of pro-inflammatory and pro-fibrotic factors. MRA acts on cardiomyocytes hypertrophy by affecting gene transcription (19). In animal study, knockdown of MR in T-cells attenuates cardiac hypertrophy. The administration of MRA in mice also blocks MR signaling, reduces oxidative stress in cardiomyocytes, inhibits inflammation and fibrosis, and reduces the extent of macrophage infiltration (20). MRAs attenuate proinflammatory molecule expression in the rat heart and subsequent vascular and myocardial damage. Thus, we can infer that finerenone treatment in rats with severe hypertension and the vascular inflammation phenotype in the heart is effective (21).

### 3.2 The mechanism of finerenone on kidney

On the kidney, MR overactivation leads to glomerular hypertrophy, sclerosis, and renal fibrosis with reduced renal blood flow, finally results in renal injury and renal dysfunction (22). Finerenone reduces the formation of damaged vascular neointima by reducing endothelial cell apoptosis and inhibiting smooth muscle cell proliferation. Finerenone can prevent adverse vascular remodeling while restoring vascular integrity, and it can also block the damage to the kidney from MR overactivation, delaying the progression of nephropathy and bringing renal benefit (23).

Finerenone reduces endothelial cell apoptosis, attenuates smooth muscle cells proliferation, and decreases leukocyte recruitment and inflammatory response after vascular injury, thereby promoting endothelial repair and preventing adverse vascular remodeling. In a mouse model of DM induced CKD, finerenone treatment shows a significant reduction of proteinuria (24). Kolkhof et al. reported that in a rat model, the expression of genes related with renal hypertrophy, proteinuria and renal inflammatory are down-regulated in finerenone-treated group compared to isodose eplerenone group (8). In addition, finerenone prevents from functional and structural heart and kidney damage in a dose-dependent manner, without affecting blood pressure. Finerenone reduced cardiac hypertrophy, plasma pro-BNP, and proteinuria more efficiently than eplerenone. In the mice model of non-diabetic nephropathy, finerenone reduces the levels of inflammatory factors, fibrogenic markers and deposition of perinephric macrophages, reduces proteinuria and tubulointerstitial fibrosis. This anti-fibrotic process is independent of blood pressure, and exhibit a dose-dependent reduction in fibroblast accumulation and collagen deposition (25). The MRA treatment also reduces glomerular pathological injury and improves renal function in glomerulonephritis mice models. In addition, finerenone treatment may also prevent ischemia-reperfusion-induced renal tubular injury (19).

In general, MR activation promotes NADPH oxidase, and enhances ROS accumulation in VSMCs and ECs. In this way, MR agonist such as aldosterone results in endothelial dysfunction, macrophage infiltration and T cell activation, inflammatory cytokines accumulation, leading to fibrosis and stiffness. MR activation aggravates podocyte damage and effacement, glomerular injury and endothelial damage, leading to vascular remodeling. MRA finerenone blocks the binding of aldosterone and MR, then attenuates those pathophysiological progressions. In this way, finerenone shows renal-cardio protective effect (**Figure 1**).

## 4 Pharmacological characteristic and safety of finerenone

Finerenone is innovative in its molecular structure. Finerenone induces MR conformational changes mainly through its side chain, leading to the prominence of helix 12 of the c-terminal, activating functional domain of the MR receptor. It affects the recruitment of co-regulatory factors and alters MR stability, nuclear translocation and activation. Finerenone has a high selectivity and innovative molecular structure compared to the first- and second-generation of MRAs.

Finerenone binds to MR with greater affinity through a large number of van der Waals forces and hydrogen bonds, has a stronger antagonistic effect, completely blocks the transcription factor aggregation caused by aldosterone-MR receptor complex, and inhibits MR overactivation. Finerenone has a short elimination half-life of only two hours. Finerenone displays shorter Tmax of 0.5~0.75h than spironolactone and eplerenone of 1-2h. Spironolactone exhibits multiple active metabolites, such as canrenone, while elperenone and finerenone has no active metabolites. Finerenone has a weak affinity for androgen and progesterone receptors. Spironolactone displays non-specific binding to steroid receptors, thus, it shows anti-androgenic effect. Compared to the first-generation MRA spironolactone, eplerenone is 40-fold less potent than spironolactone. However, eplerenone and those non-steroidal MRAs (including finerenone, esaxerenone, ocedurenone, balcinrenone, ect.) generally display exhibits greater selectivity for MR over other steroid hormone receptors. Therefore, finerenone shows less risk of sex hormone related adverse effects (26).

Finerenone shows similar potency to spironolactone, but high affinity to MR. Compared to the previous steroidal MRAs spironolactone or eplerenone, the new-generation MRA finerenone shows its superiority (**Table 1**). The side effects including sex hormone associated adverse effects, risk of hyperkalemia and renal insufficiency are lower in finerenone (26). It is safer and more favorable for T2DM patients’ treatment. Finerenone also inhibits the expression of downstream pro-inflammatory and pro-fibrotic factors, providing effective anti-inflammatory and anti-fibrotic effects.

**Table 1:** The Pharmacokinetics of MRAs.

Agent	Spironolactone	Eplerenone	Finerenone	Esaxerenone	Apararenone	Ocedurenone	Miricorilant	Balcinrenone	Drospirenone/Estetrol	Canrenone
**Name**	SC 9420		BAY 94-8862	CS-3150	MT-3995	KBP-5074	CORT-118335	AZD-9977		SC 9376
**Dose**	10mg/20mg	25mg/50mg	10mg/20mg	1.25mg/2.5mg/5mg	2.5mg/5mg/10mg	0.25mg/0.5mg	in development	in development		
**Company**	Pfizer	Pfizer	Bayer	Daiichi-Sankyo Company Limited, Japan	Mitsubishi Tanabe Pharma Corporation	KBP Biosciences	Corcept, Argenta Discovery	AstraZeneca	Mithra Pharmaceuticals, Estetra S.A., Libbs	
**Generation of MRA**	first	second	third	third	MRA	MRA	GRA; MRA	MR regulator	MRA; PR agonist; ARA; selective ER regulator	first
**Steroidal/Nonsteroidal**	steroidal	steroidal	nonsteroidal	nonsteroidal	nonsteroidal	nonsteroidal	nonsteroidal	nonsteroidal		steroidal
**Molecular Formular**	C24H32O4S	C24H3O6	C21H22N4O3	C22H21F3N2O4S	C17H17FN2O4S	C28H30ClN5O2	C24H23F3N2O2	C20H18FN3O5	C24H30O3	C22H28O3
**Structure**	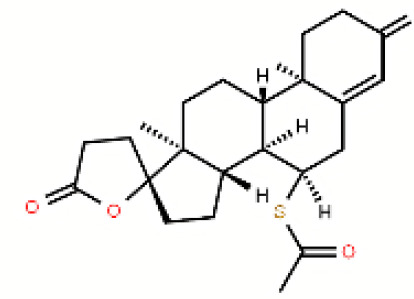	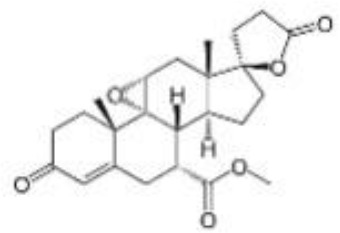	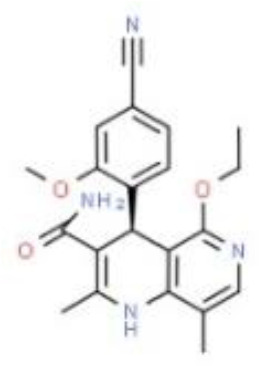	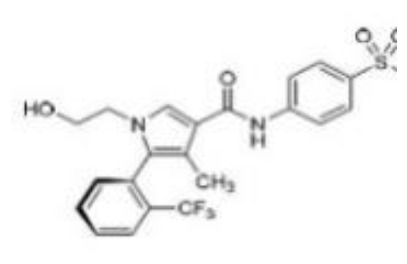	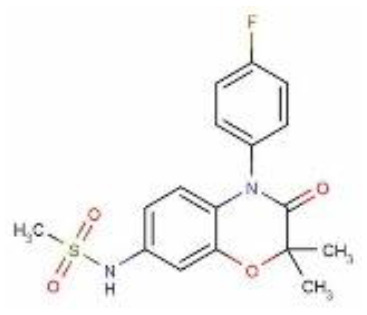	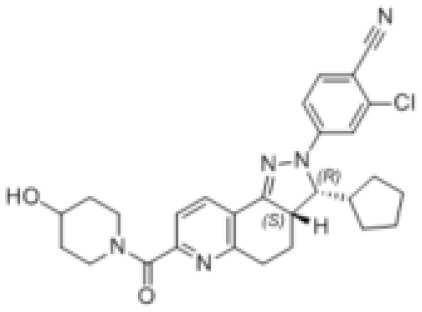	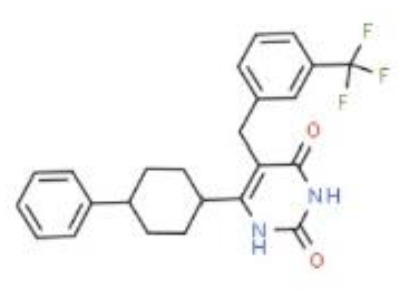	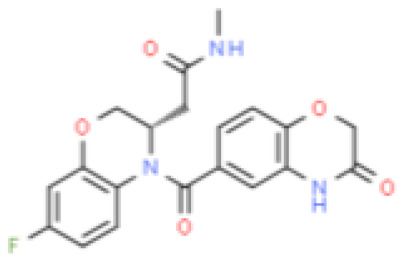	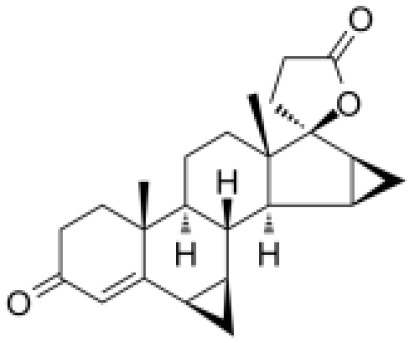	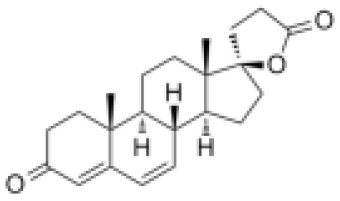
**Characteristic**	potent and unselective	less potent and more selective than spironolactone	more potent and more selective than spironolactone	more potent and more selective than eplerenone	more potent and more selective than spironolactone		more potent and more selective; moderate affinity to MR	no affinity to GR/PR/AR		
**Heart-kidney distribution ratio**	1:6	1:3	1:1; cannot across the Blood-Brain Barrier	1:1						
**t½**	13~24h(qd/bid); 9~16hqid	4-6h	1.7-2.8h	20-30h	275-285h for parent drug;>1000h for active metabolite			increased with dose	31h	
**Tmax**	2.6-3.05h	1.5h	0.75-1h	1.5-4h	4h			0.5-0.8h	1-2h	
**Cmax**	209-301ng/ml	–	160ug/L(20mg)						37ng/ml	
**MR IC50**	24	990	17.8				moderate affinity to MR			
**AR IC50**	77	≥21240	≥10000					almost no affinity to GR/PR/AR	almost no affinity to GR/AR	
**GR IC50**	2410	≥21980	≥10000							
**PR IC50**	740	≥31210	≥10000							
**Oral bioavailability**	>90%	69%	86.50%						76-85%	
**Protein binding ratio**	>90%	33%-60%	92%						95-97%	
**Metabolism**	prodrug with multiple active metabolites	no active metabolites	no active metabolites	n/a	metabolite with low activity (MR binding affinity one-fiftieth of that of apararenone)			n/a		the active metabolites of spironolactone
**Hyperkalemia**	high risk	high risk	low risk	low risk					no risk	
**Excretion**	<1% unchanged drug recovered in urine; 10-15% of dose excreted in urine form of metabolites	66% of dose excreted via urine; <3% unchanged drug recovered from urine	80% of dose excreted via urine; <1% unchanged drug excreted in urine	38.5% of dose excreted in urine; <2% unchanged drug excreted in urine	<14% of dose excreted in urine			24–37% of dose excreted in urine; 20% of dose excreted unchanged in urine		
**Sex-like ADR**	common	less than spironolactone	no statistics difference with placebo group							
**Dose adjust based on renal function**	excretion through the kidney	cannot be removed by hemodialysis	decrease dose in patients with eGFR≤60 and prohibit when eGFR<25						decrease dose in patients with eGFR 30-50	
**Indication**	PA; HBP; hypokalemia; edema; HF	congestive heart failure; HBP	T2DM with CKD, ESRD, CVD, congestive heart failure	HBP, DKD(clinical trial phase)	DKD	HBP, DKD, HN	obesity; prostate cancer; metabolism disorder	DKD	contraception	HBP
**Contraindication**	not recommended to CRF	not recommended to CRF								

The unit of IC50 is nmol/L. the unit of eGFR is ml/min/1.73m^2^. PR, progesterone receptor. ER, estrogen receptor. AR, androgen receptor. MRA, mineralocorticoid receptor antagonist. t½, geometric mean terminal half-life. Tmax, median time to maximum plasma concentration. Cmax, maximum plasma concentration. ADR, adverse drug reactions. eGFR, estimated glomerular filtration rate. PA, primary aldosteronism. HBP, hypertension. CKD, chronic kidney diseases. DKD, diabetic kidney diseases. HN, hypertensive nephropathy. ESRD, end-stage renal disease.

Finerenone, the new oral MRA is a kind of naphthyridine derivatives based on dihydropyridines (DHP) structure, inhibits MR activation precisely and potently, and show stronger anti-inflammatory and anti-fibrotic effects than the first- and second-generation of steroidal MRAs. Finerenone is balanced distributed in heart and kidney (8), therefore, it has cardio-renal double benefits. While spironolactone and eplerenone mainly distributes in the kidney (19). Otherwise, finerenone is not allowed to across the blood-brain barrier (BBB). Quantitative whole-body autoradiography with [14C]-labeled finerenone does not demonstrate in brain (27). Both finerenone and spironolactone act as affecting the transcriptional process. Spironolactone inhibits the binding of cortisol to the receptor while also acting as a partial agonist. However, finerenone acts as an inverse agonist after binding to the promoter, reducing the activation of a kind of transcriptional cofactors (SRC-1) and inhibiting the transcriptional process even in the absence of aldosterone (28). In phase II trials, the novel MRAs have comparable efficacy compared to the conventional MRAs, but exhibiting a significant safety profile in patients with HF and renal dysfunction (29).

In the safety analysis of FIDELITY study, the discontinuation of finerenone associated hyperkalemia is low, and is comparable to placebo (30). In the FIDELITY study, the incidence of treatment-emergent adverse events (TEAE) is similar in the finerenone and placebo groups, with no increase in sex hormone-related side effects compared to the placebo group. In addition, finerenone has no effect on glycated hemoglobin A1c (HbA1c). In terms of CKD stage, the safety profile of finerenone in T2DM patients with CKD stage 4 is consistent with that of CKD stages 1 to 3 (31). In the FIDELIO-DKD study, emergency adverse events, diarrhea, nausea, vomiting, and hypovolemia are analyzed, and those adverse events in the treatment group are similar to placebo group. The conventional steroidal MRAs have limited long-term usage due to the potential adverse effects such as hyperkalemia, but the advent of finerenone shows a promising direction for the treatment of T2DM with CKD and CVD (32).

## 5 Effect of finerenone on cardiovascular disease outcomes in T2DM

Clinical studies show the protective effect of finerenone on cardiovascular outcomes to provide evidence (**Table 2**). Both the Efficacy and Safety of Finerenone in Subjects With Type 2 Diabetes Mellitus and the Clinical Diagnosis of Diabetic Kidney Disease (FIGARO-DKD) and Efficacy and Safety of Finerenone in Subjects With Type 2 Diabetes Mellitus and Diabetic Kidney Disease (FIDELIO-DKD) studies are large-scaled multicenter phase III clinical studies, focusing on the effect of finerenone on the composite cardiovascular-renal outcomes as the primary endpoints in T2DM patients with CKD (3, 6, 26). The findings of FIGARO-DKD study ultimately show 13% reduction in cardiovascular composite endpoint events (including cardiovascular death, nonfatal myocardial infarction, nonfatal stroke, or hospitalization for HF) with finerenone in T2DM patients with CKD. In the patients with well-controlled blood pressure and glycemic levels, or using a combination of RAAS inhibitors, finerenone also shows the consistent results (6). There was no significant difference in safety from placebo. The results of the FIDELIO-DKD study shows that finerenone significantly reduce the risk of composite cardiovascular outcomes compared with placebo, with no significant difference in outcomes in patients with or without established ASCVD. Furthermore, the rate of treatment discontinuation due to hyperkalemia is low (26). The FIDELITY study is the meta-analysis study based on the FIDELIO-DKD study and the FIGARO-DKD study. The results of the study show that finerenone significantly reduce the risk of cardiovascular composite endpoint events in patients with T2DM with CKD by 14% (HR=0.86; 95% CI: 0.78-0.95; P=0.0018) compared to placebo independent of established ASCVD. The risk of HF associated hospitalization is significantly reduced by 18% (RR=0.82; 95% CI: 0.71-0.94), and the risk of all-cause mortality is reduced by 15% (RR=0.85; 95% CI: 0.74-0.99) (31, 61).

**Table 2:** The Trials Information of MRAs.

Drug	Trial	Characteristic	n	Groups	Median Follow-up	Inclusion criteria	Outcome	Conclusion
**Spironolactone**								
SPI	RALES (Pitt et al.) (33)	a double-blind RCT	1663	standard therapy and SPI or placebo	24 months	severe HF with LVEF≤35%	death from all causes	SPI, in addition to standard therapy, substantially reduces the risk of both morbidity and death among patients with severe HF.
SPI	Tseng et al. (Taiwan National Health Insurance Research Database) (34)	retrospective cohort study	27213	SPI usage +/-before	3-4 years	CKD stage 5	all-cause mortality, HHF and MACE (the composite of AMI and ischemic stroke)	SPI may be associated with higher risks for all-cause and infection-related mortality and HHF in pre-dialysis stage 5 CKD patients.
SPI	Yang et al. (primary date from Taiwan's National Health Insurance Research Database) (35)	retrospective cohort study	2079	SPI usage +/-before	\	CKD stage 3-4	ESRD, MACE, HHF, HKAH, all-cause mortality and CV mortality	SPI represented a promising treatment option to retard CKD progression to ESRD amongst stage 3–4 CKD patients, but strategic treatments to prevent hyperkalemia should be enforced.
SPI	TOPCAT(NCT00094302) (36)	RCT(phase III)	3445	SPI vs. placebo	6 years	HFpEF (symptomatic, HHF within the past year)	composite outcome of CV mortality, aborted cardiac arrest, or HHF	SPI does not significantly reduce the incidence of the primary composite outcome of death from CV causes, aborted heart arrest or HHF in patients with HFpEF. Greater potassium and creatinine changes and possible clinical benefits with SPI in patients with HFpEF from the Americas.
SPI	Enzan et al. (Japanese Cardiac Registry of Heart Failure in Cardiology database) (37)	retrospective registration study	457	SPI usage +/-before	2.2 years	HFmEF (LVEF 40%-49%)	a composite of all-cause death or HHF	Among patients with HHF for HFmEF, SPI shows better long-term outcomes.
SPI	Krieger et al. (NCT01643434) (38)	multicenter RCT study	1597	SPI vs. clonidine	3 months	resistant hypertension	BP control during office (<140/90 mm Hg) and 24h ambulatory (<130/80 mm Hg) BP monitoring	SPI promotes greater decrease in 24h systolic and DBP and diastolic daytime ambulatory BP than clonidine
**Eplerenone**								
EPL	Minakuchi et al.(UMIN000008521) (39)	a single-blinded placebo-controlled prospective observational study	48	ACEI/ARB + EPL or placebo	24-36 months	patients with CKD stage 2-3 whose plasma ALD concentration was above 15 ng/dL	change in eGFR	MRA can be an effective in preventing CKD progression, especially in patients with high plasma ALD.
EPL	ElMokadem et al.(NCT04143412) (40)	a single-blind RCT	75	ramipril or EPL or both	24 weeks	T2DM+hypertension and DKD (microalbuminuria)	BP, UACR, serum creatinine, eGFR and serum K level	Addition of EPL to ACEI shows an added anti-albuminuria effect without significant change of the serum potassium level compared with EPL or ACEI.
EPL	EPOCH(NCT01832558) (41)	a exploratory RCT study	15	ACEI + EPL or placebo	10 weeks	CKD stages 2-3 and albuminuria due to DKD	quantify plasma angiotensin levels, renin and ALD in PA for 8 weeks MRA treatment.	Combined EPL and ACEI therapy increases Ang-(1–7) levels in patients with CKD indicating a unique nephroprotective RAAS pattern with considerable therapeutic implications.
EPL	EPHESU (42)	a multicenter, international, double-blind, phase III RCT	6442	different dosage of EPL vs. placebo	16 months	AMI after 3-14 days with HFrEF (LVEF≤40%)	death from any cause and death from CV causes or HHF, AMI, stroke, or ventricular arrhythmia	The addition of EPL to optimal medical therapy reduces morbidity and mortality among patients with AMI complicated with HFrEF.
EPL	EMPHASIS-HF(NCT00232180) (43)	a double-blind phase III RCT study	2737	different dosage of EPL vs. placebo	21 months	HFrEF (NYHA II) with LVEF≤35%	a composite of death from CV causes or HHF	EPL reduces both the risk of death and the risk of hospitalization among patients with systolic HF and mild symptoms.
EPL	RAAM-PEF(NCT00108251) (44)	a double-blind, placebo-controlled RCT	44	EPL vs. placebo	6 months	HFpEF and hypertension with/without T2DM	changes in 6-minute walk distance, diastolic function, and biomarkers of collagen turnover	EPL is associated with significant reduction in markers of collagen turnover and improvement in diastolic function.
EPL	Schneider et al.(NCT00138944) (45)	a double-blind, placebo-controlled, parallel group RCT	51	regular BP medication + low dosage EPL or placebo	6 months	TRH	LVM assessed by MRI before and after treatment	MRA should be used preferentially in patients with TRH in order to achieve an effective reduction of LVM along with the improvement of BP control.
EPL	Kalizki et al.(NCT00138944) (46)	double-blinded, placebo-controlled parallel-group RCT	51	regular BP medication + low dosage EPL or placebo	6 months	TRH	vascular parameters including PWV, AIx, AP, AP@HR75, RRI, IMT and UAER	EPL beneficially affects markers of arterial stiffness and wave reflection in patients with TRH, independently of BP lowering.
EPL	OWASE(UMIN000005956) (47)	a multicenter, prospective, open-label RCT	195	EPL vs. thiazide diuretic	48 weeks	ARB-treated hypertension and albuminuria	the change of UACR from baseline to 48 weeks	The antialbuminuric effects and safety of EPL therapy are similar to thiazide diuretics when combined with ARBs in patients with hypertension and albuminuria.
EPL	Karashima et al.(UMIN000004581) (48)	an open-label, non-controlled, prospective cohort study	54	EPL vs. SPI	12 months	PA	metabolic factors including BMI, HOMA-IR, serum creatinine, potassium and lipids, UAE and PAC and PRA	EPL and SPI decreases BP and increases serum potassium levels to similar degrees. PAC and PRA are similar between the two groups.
EPL	EPATH(NCT02136771) (49)	RCT and observational data prospective cohort study	4	different dosage EPL vs. placebo	8 weeks	PA	ARR	MRA does not significantly alter the ARR in primary hyperparathyroidism patients but significantly reduces the ARR in PA patients.
**Finerenone (BAY 94-8862)**								
FIN	ARTS(NCT01807221; NCT01874431) (7)	multicenter, parallel-group, phase II study, with double-blind placebo and open-label SPI comparator arms phase II RCT	458	standard therapy and different dosage (2.5mg/5mg/10mg qd) FIN or placebo	about 30 days	HFrEF (NYHA II-III, LVEF≤40%) and mild or moderate CKD (eGFR 60 to <90 and 30-60 mL/min/1.73 m2, respectively)	serum potassium concentration, eGFR, and albuminuria	In patients with HFrEF and moderate CKD, BAY 94-8862 5–10 mg/day was at least as effective as SPI 25 or 50 mg/day in decreasing biomarkers of hemodynamic stress, but it was associated with lower incidences of hyperkalemia and WRF.
FIN	ARTS-HF(NCT01807221) (50)	a double-blind placebo and open-label SPI comparator arms phase IIb RCT study	1066	different dosage FIN vs. EPL	90 days	worsening CHF with exasperated HFrEF and CKD and/or T2DM requiring hospitalization and intravenous diuretic therapy	the percentage of participants with a relative decrease in NT-proBNP of more than 30% from baseline to day 90	FIN is well tolerated and induced a 30% or greater decrease in NT-proBNP levels in a similar proportion of patients to EPL.
FIN	ARTS-DN(NCT01874431) (51)	a multicenter, double-blind, placebo-controlled, parallel-group phase II RCT	823	ACEI/ARB + different dosage FIN or placebo	90 days	T2DM with DKD (albuminuria)	ratio of UACR at day 90 to UACR at baseline	Among patients with DN, most receiving an ACEI/ARB, the addition of FIN compared with placebo resulted in improvement in the UACR.
FIN	FIDELIO-DKD(NCT02540993) (52)	a double-blind, placebo-controlled, parallel-group, multicenter, event-driven phase III study	5734	ACEI/ARB + FIN (10mg/20mg qd) or placebo	32 months (2.6 years)	T2DM with DKD	the first occurrence of the composite endpoint of onset of kidney failure, a sustained decrease of eGFR ≥40% from baseline over at least 4 weeks, or renal death	In patients with CKD and T2DM, FIN lowers the risks of CKD progression and CV events than placebo.
FIN	FIGARO-DKD(NCT02545049) (3)	a double-blind, placebo-controlled, parallel-group, multicenter, event-driven phase III study	7337	standard therapy + FIN or placebo	41 months (3.4 years)	T2DM with DKD	the first occurrence of the composite endpoint of CV death, non-fatal myocardial infarction, nonfatal stroke, or HHF	T2DM and stage 2 to 4 CKD with moderately elevated albuminuria or stage 1 or 2 CKD with severely elevated albuminuria, FIN therapy improved CV outcomes as compared with placebo.
FIN	FIDELITY(NCT02540993; NCT02545049) (31)	meta analysis (FIDELIO-DKD and FIGARO-DKD)	13026	standard therapy + FIN or placebo	2.3-3.8 years	T2DM with DKD	a composite of CV death, non-fatal MI, non-fatal stroke, or HHF, and a composite of kidney failure, a sustained ≥57% decrease in eGFR from baseline over ≥4 weeks, or renal death	FIN reduces the risk of clinically important CV and kidney outcomes vs. placebo across the spectrum of CKD in T2DM
FIN	FINEARTS-HF(NCT04435626)	a double-blind, placebo-controlled, parallel-group, multicenter phase III Study	5500	different dosage of FIN	–	HFmEF (LVEF≥40%) with clinical symptom	number of CV deaths and HF events	ongoing
FIN	FIND-DKD(NCT05047263)	a randomized, double-blind, placebo-controlled, parallel-group, multicenter phase III study	1500	FIN vs. placebo	–	CKD without T2DM	change of the slope of eGFR	ongoing
FIN	CONFIDENCE(NCT05254002)	a parallel-group treatment, phase II, double-blind, three-arms study	807	FIN+empagliflozin vs. FIN+placebo vs. empagliflozin+placebo	180-210 days	CKD with T2DM	relative changes from baseline in UACR at 180 days in combination therapy group versus empagliflozin/FIN alone	ongoing
FIN	Fu et al. (53)	meta-analysis	7048	standard therapy + FIN or placebo	–	DM patients with CKD (phase 2)	assessed at least one of the following outcomes: UACR, eGFR, adverse events including CV disorders and hyperkalemia	FIN confers an important antiproteinuric effect on patients with CKD and reduces the risk of CV disorders
FIN	Pei et al. (54)	meta-analysis	1520	FIN vs. SPI vs. EPL	–	CHF with HFrEF	effective number of cases with a 30% reduction in NT-proBNP	FIN reduces NT-proBNP, UACR, and other biochemical indicators in a dose-dependent manner.
**Esaxerenone (CS-3150)**								
ESA	ESAX-HTN(NCT02890173) (55)	double-blind, three parallel placebo comparator arms phase III trial	1001	different dosage of ESA or EPL	12 weeks	essential hypertension	changes in SBP/DBP at rest relative to baseline after 12 weeks	ESA is an effective and well-tolerated MRA in Japanese patients with essential hypertension, with BP-lowering activity at least equivalent to EPL.
ESA	ESAX-DN(JapicCTI-173695) (56)	multicenter, double-blind, placebo control, two- arm, parallel group, comparison study	449	CS-3150 vs. placebo	52 weeks	T2DM with microalbuminuria taking ACEI/ARB	UACR remission rate at the end of the treatment	Adding ESA to existing RAAS inhibitors therapy in patients with T2DM and microalbuminuria increased the likelihood of albuminuria returning to normal levels, and reduced progression of albuminuria to higher levels.
**Apararenone (MT-3995)**								
APA	Izumi et al.(NCT02517320; NCT02676401) (57)	a double-blind, placebo-controlled study	293/241	different dosage of APA vs. placebo	24-52 weeks	stage 2 diabetic nephropathy (DN)	the 24-week percent change from baseline in UACR and 24- and 52-week UACR remission rates	The UACR-lowering effect of APA administered once daily for 24 weeks in patients with stage 2 DN was confirmed, and the 52-week administration was safe and tolerable.
**Ocedurenone (KBP-5074)**								
OCE	BLOCK-CKD(NCT03574363) (58)	a double-blind, placebo-controlled, global, multicenter phase IIb trial	162	different dosage of APA vs. placebo	12 weeks	moderate-to-severe (stage 3b/4; eGFR 15-44 mL/min/1.73m^2^) CKD and uncontrolled hypertension	changes in trough-cuff seated SBP/DBP and UACR from baseline to day 84	KBP-5074 demonstrated a clinically meaningful trend in the reduction of UACR.
OCE	CLARION-CKD(NCT04968184)	a phase 3 double-Blind placebo-controlled multicenter study	600	OCE vs. placebo	52 weeks	uncontrolled hypertension and moderate or severe CKD (stage 3b/4)	changes in seated trough-cuff SBP from baseline to week 12/48/52	ongoing
**Canrenone**								
CAN	COFFEE-IT(NCT03263962) (59)	a multicenter, retrospective, observational study	532	treated +/- CAN	10 years	CHF with HFpEF (LVEF ≥ 50%)	the rate of CV mortality in CHF and the rate of death and survival.	CAN preserves systolic fraction, reduces mortality and extends life in CHF patients.
CAN	AREA-in-CHF(NCT00403910) (60)	RCT (phase 3)	500	CAN vs. placebo	12 months	compensated HFrEF with LVEF≤45%	changes in echocardiographic left ventricular diastolic volume	CAN, with optimal therapy (ACEI/ARB, β-blockers) in patients with metabolic syndrome, stabilized HF with reduced EF, protects deterioration of myocardial mechano-energetic efficiency, improves diastolic dysfunction and maximizes the decrease in BNP.

ALD, aldosterone. SPI, spironolactone. EPL, eplerenone. FIN, finerenone. ESA, esaxerenone. APA, apararenone. OCE, ocedurenone. CAN, canrenone. RCT, randomized controlled trials. MACE, major adverse cardiovascular events. ESRD, end-stage renal disease. HHF, hospitalization for heart failure. HKAH, hyperkalemia-associated hospitalization. CV, cardiovascular. CHF, chronic heart failure. HF, heart failure. HFmEF, heart failure with mild ejection fraction. HFrEF, heart failure with reduced ejection fraction. HFpEF, heart failure with preserved ejection fraction. NT-proBNP, amino-terminal pro-B-type natriuretic peptide. AMI, acute myocardial infarction. LVM, left ventricular mass. BP, blood pressure. SBP, systolic blood pressure. DBP, diastolic blood pressure. TRH, treatment-resistant hypertension. CKD, chronic kidney diseases. DKD, diabetic kidney diseases. T2DM, type-2 diabetic mellitus. DN, diabetic nephropathy. eGFR, estimated glomerular filtration rate. UACR, urinary albumin-to-creatinine ratio. MRA, mineralocorticoid receptor antagonists. RAAS, renin-angiotensin-aldosterone system. ACEI, angiotensin-converting enzyme inhibitors. ARB, angiotensin II receptor blockers. PA, primary aldosteronism. ARR, aldosterone to renin ratio. PAC, plasma aldosterone concentration. DRC, direct renin concentration. PWV, pulse wave velocity. AIx, augmentation index. AP, augmentation pressure. AP@HR75, AP normalized to a heart rate of 75/min. RPI, renal resistive index. IMT, intima-media thickness. UAER, and urinary albumin excretion rate. BMI, body mass index. HOMA-IR, homeostasis model assessment-insulin resistance. UAE, urinary albumin excretion. PAC, plasma aldosterone concentration. PRA, plasma renin activity. WRF, worsening renal failure. NCT, the number of trials in ClinicalTrials.gov. UMIN, the number of trials in UMIN Clinical Trials Registry (UMIN-CTR); JapicCTI, the number of trials in JAPIC Clinical Trials Information.

## 6 Effects of finerenone on renal outcomes in T2DM patients with CKD

MR overactivation is one of the key pathophysiological mechanism in patients with T2DM and CKD. The inflammatory and fibrotic mediated effects occur when MR is overactivated, leading to the progression of CKD (62). Finerenone reduces the urinary albumin-to-creatinine ratio (UACR) in T2DM with CKD patients (63). Proteinuria is also an independent predictor of CVD risk. Elevated proteinuria portends pre-existing endothelial damage (64). Overactivation of MR is an important mechanism leading to endothelial damage (**Figure 1**). Thus inhibition of MR overactivation is essential to suppress endothelial injury, reduce CV risk, and delay progression of CKD (65).

FIGARO-DKD and FIDELIO-DKD are two large-scaled phase III clinical studies (**Table 2**), involving T2DM with CKD patients, and the endpoints are cardiorenal outcomes. In the FIGARO-DKD study, finerenone significantly reduces renal composite endpoint events (occurrence of renal failure, sustained decline in eGFR ≥57% from baseline, or death from renal disease) by 23% (6). The results of the FIDELIO-DKD study show that finerenone significantly reduces the risk of renal composite endpoint events by 18% compared with placebo on the basis of standard treatment (HR=0.82; 95% CI[0.73-0.93]; P=0.0001) (52). Finerenone reduces albuminuria in short-term intervention involving T2DM patients with CKD. However, the long-term effects on renal and cardiovascular outcomes are unknown. Finerenone reduces the risk of major outcome events including renal failure, 40% reduction in eGFR or death due to renal diseases, while the adverse events are comparable to placebo group (52).

The results of the FIDELITY study, a pooled analysis of the FIDELIO-DKD and FIGARO-DKD studies, show that finerenone significantly reduce the risk of renal composite events by up to 23% (HR=0.77; 95% CI: 0.67-0.88; P=0.0002) and significantly reduced UACR by 32% compared to placebo. Further analysis reveals that finerenone decreases the incidence of all non-lethal renal outcomes, including end-stage renal disease (ESRD). Finerenone reduces cardiovascular risk in T2DM patients with CKD in all UACR and eGFR stages (51, 66). Finerenone significantly reduced the risk of renal composite events by 29% (RR=0.71; 95% CI: 0.57-0.88) in patients with established ASCVD and by 19% in patients without ASCVD history compared with placebo (RR=081; 95% CI: 0.68-0.97). The renal benefit of finerenone and the effect of reducing all-cause mortality are not affected by ASCVD history (67).

## 7 Finerenone in combination therapy with ACEI/ARB and SGLT-2i/GLP-1RA

Finerenone therapy improved cardiovascular and kidney outcomes in the FIDELITY pooled analysis (66). SGLT-2i and GLP-1RA can also improve cardio-renal endings independently, which play a significant role in inhibiting fibrillation, reducing urine protein, controlling inflammation, anti-oxidative stress and delaying atherosclerosis (68). However, the effect and mechanism on combination with ACEI/ARB and SGLT-2i/GLP-1RA, the established cardio-renal protective anti-hypertension or anti-diabetic agents, are unclear. Finerenone combines with either SGLT-2i/GLP-1 RA may enhance the effect of anti-inflammation, anti-oxidative stress, and endothelial protection. Whereas, clinical trials and deep mechanism research are needed to provide evidence. A meta-analysis based on the combination therapy of oral glycemic-lowering agents with finerenone included FIDELIO-DKD and FIGARO-DKD, with the primary outcomes of MACE events, illustrates that finerenone does not significantly increase cardiovascular benefit in T2DM patients with “add-on” the SGLT-2i or GLP-1RA, but confirms the significant efficacy of single-agent finerenone in cardio-renal improvement (69). It provides a basis for guiding clinical use. However, the evidence may be not conclusive due to the limited number of RCTs.

In the subgroup analysis from FIDELIO-DKD trial, finerenone reduces UACR by 31% in patients with or without GLP-1RA usage at baseline. It suggests that finerenone improve the kidney and CV outcomes independent of GLP-1RA use (66). It suggests the renal-protective effect of finerenone in patients already treated with GLP-1RA and demonstrates that GLP-1RA is also a UACR-reducing treatment since previous meta-analysis has shown that GLP-1RA is marginally reduced UACR (70, 71). Animal studies clarify that the combination of finerenone and SGLT-2i provides renal protection effect in a mouse model of hypertension-induced cardiorenal disease. The combination administration significantly reduces proteinuria levels in mice compared to single agents (72). However, several clinical studies, including FIDELIGO-DKD, have shown that finerenone alone reduces UACR independent of SGLT-2i, and similar in heart failure with reduced ejection fraction (HFrEF) patients, SGLT-2i alone significantly improves cardiovascular outcomes, even without finerenone (73).

The CONFIDENCE study (A Study to Learn How Well the Treatment Combination of Finerenone and Empagliflozin Works and How Safe it is Compared to Each Treatment Alone in Adult Participants With Long-term Kidney Disease and Type 2 Diabetes, NCT05254002) is an ongoing randomized controlled study of the efficacy of finerenone in combination with SGLT-2i Empagliflozin in T2DM patients with CKD. Both finerenone and empagliflozin are guideline-recommended clinical agents for the treatment of DKD. The study was designed to investigate whether the two-agents combination is superior to monotherapy, focusing on the endpoints of UACR, eGFR change and incidence of hyperkalemia. Clinical evidence of the additional benefit of finerenone in combination with empagliflozin will be available at the end of the study (74). The analysis of the CONFIDENCE study, which is scheduled to end in May 2023, include both the combination group and the monotherapy group of empagliflozin and finerenone. Thus, the analysis of this study may provide stronger evidence to show whether the combination is superior to monotherapy for clinical use.

Another clinical study investigates the effect of finerenone on proteinuria in DKD patients, with the treatment combination with renin-angiotensin-aldosterone system (RAAS) inhibitors (ACEI/ARB) for 90 days. The results show a significant and dose-dependent improvement of UACR in all dose-groups of finerenone compared to the placebo group (51). A meta-analysis of combination therapy for DKD show that the combination of MRA with ACEI/ARB further reduce the urine albumin excretion rate (UAER) compared with ACEI/ARB monotherapy. eGFR is not statistically different between the two groups, but the serum creatinine level is significantly increased in the combination group. A subgroup analysis based on different MRAs yields that the relative risk of hyperkalemia with the ACEI/ARB combination with finerenone is lower than with eplerenone or spironolactone (75).

In summary, either finerenone alone or in combination with SGLT-2i or GLP-1RA may improve DKD outcomes and risk of cardiovascular events in T2DM patients, but the results of clinical studies for combination versus monotherapy varies. More clinical trials are needed to provide conclusive evidence. The deep-insight of molecular mechanism and the cross-talk links among finerenone and ACEI/ARB and SGLT-2i/GLP-1RA agents unclear. Thus, further basic studies are excepted.

## 8 Prospects for finerenone treatment

In clinical observation, finerenone show potential therapeutic effects in diabetic retinopathy (DR). A phase III clinical trial ReFineDR (NCT04477707)/DeFineDR (NCT04795726) on the effect of finerenone on slowing the progression of non-proliferative diabetic retinopathy (NPDR) is currently ongoing. A total of 244 patients with DR at baseline (134 in the finerenone group and 110 in the placebo group) are enrolled from the FIDELIO-DKD or FIGARO-DKD studies to investigate, with the primary outcome of the NPDR progression. At baseline, most patients had mild-to-moderate NPDR. After two-year observation, 3.7% and 6.4% of patients in the finerenone and placebo groups, respectively, show vision-threatening events, and fewer participants in the finerenone group require ocular intervention (76). The results of this trial are pending and the data are continuously being updated.

In the FIGARO-DKD study, HF or exacerbation of HF causing death as endpoints, finerenone reduces the risk of new-onset HF and improves exacerbation of HF in T2DM patients with CKD, regardless of the prior HF history (6). In the FIDELIO-DKD study, finerenone reduces the risk of new-onset atrial flutter or atrial fibrillation (AFF) in T2DM patients with CKD and T2DM, regardless of the AFF history at baseline (77).

Both pre-clinical and clinical studies support the correlation between increase adiposity and MR activation. In an cohort analysis, obesity was correlated with elevated aldosterone levels (78). In animal study, finerenone improves metabolic parameters, including the glucose-lipid metabolism and insulin resistance in high-fat-diet mice. Finerenone stimulates the brown adipose tissue function, and increases the expression of uncoupling protein-1 (UCP-1) through AMP-activated protein kinase (AMPK)-UCP-1 pathway (79). The effect of finerenone on anti-obesity and regulation of metabolic parameters needs more clinical evidence.

Primary hyperaldosteronism (PA) is a common cause of secondary increased hypertension, which also acceleration the progression of cardiovascular complication. Unilateral adrenal hyperplasia or adenoma is first-line treated by surgery, while bilateral adrenal hyperplasia or idiopathic hyperaldosteronism is treated by MRAs (80). Spironolactone and eplerenone are commonly recommended choice at present. Finerenone as a new MRAs, may have more prominent advantages in the treatment of PA. Further clinical studies on this agent will provide supportive evidence.

Obstructive sleep apnea hypopnea syndrome (OSAHS) is considered as an independent risk factor for hypertension, and the pathophysiological mechanisms include RAAS activation, oxidative stress, endothelial cell damage, and sympathetic nerve excitation. The elevated aldosterone levels can increase nocturnal fluid transfer, and aggravate OSAHS. There is an interaction between OSAHS and aldosterone, which aggravate the occurrence of hypertension in OSAHS patients. MRA can improve the control of hypertension and delay the development of OSAHS. Therefore, the application of aldosterone receptor antagonist can improve OSAHS related Hypertension. Finerenone as a novel MRA also may be a promising therapeutic strategy of OSAHS and OSA-related hypertension (81).

A rat experiment demonstrates that MR is overexpressed in experimental and human pulmonary arterial hypertension (PAH), with the monocrotaline and sugen/hypoxia rat models. In addition, hMR+ (human MR overexpressing) mice display increased right ventricular systolic pressure, right ventricular hypertrophy, and remodeling of pulmonary arterioles. Finerenone-feeding mice show reversed PAH in some extent and decreased inflammatory cell infiltration and vascular cell proliferation. This experiment confirmed that finerenone appears to a potential therapy for PAH (9).

## 9 Summary

Finerenone is marketed as the first third-generation highly selective non-steroidal MRA for improving cardiorenal prognosis in T2DM patients with CKD and CVD. Several large-scaled clinical trials show that, regardless of ASCVD history, finerenone reduces the risk of cardiovascular and renal adverse events in T2DM patients, delays the disease progression and improves cardiac and renal outcomes. Improving cardiorenal outcomes and delaying the progression of complications are the vital strategy for diabetic management. The integrated management of diabetic-cardio-renal contributes to long-term prognosis of diabetic patients, especially combined with CKD and CVD. Finerenone provides organ protection, also has a lower incidence of electrolyte disturbances such as hyperkalemia than those conventional MRAs due to its high selectivity and affinity to MR. There is potential effect on the treatment of primary aldosteronism (PA), diabetic retinopathy (DR), atrial fibrillation and pulmonary hypertension. It suggests that finerenone may be a potential therapeutic strategy for treatment of CKD and CVD.

## Author contributions

RL and BD drafted the manuscript. LC, SL, and YW provided helpful suggestions. LX conceived the study. BD designed the study and take responsibility for this study. All authors contributed to the article and approved the submitted version.
